# Supplementary figures and images for: FGMP: assessing fungal genome completeness
Source: BMC Bioinformatics. 2019 Apr 15;20:184. doi: 10.1186/s12859-019-2782-9 (PMC6466665; doi:10.1186/s12859-019-2782-9)

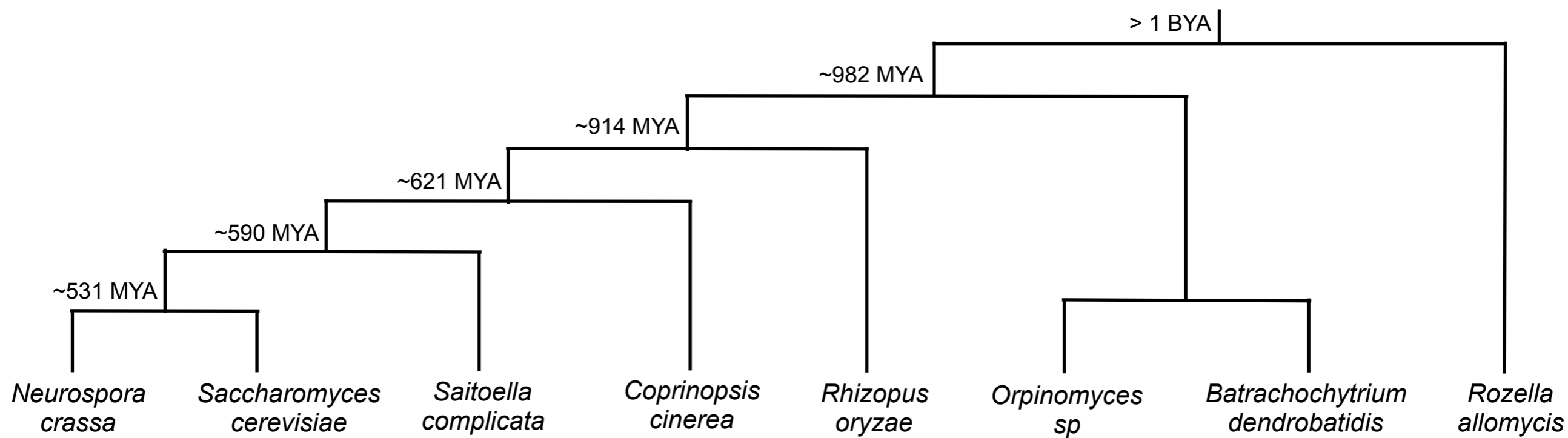

Supplement: Supplementary file 2 — Phylogeny of nine fungal species used for the detection of highly conserved nucleotide elements. The divergence times were obtained from http://www.timetree.org [30] (PDF 25 kb) [file 12859_2019_2782_MOESM2_ESM.pdf]

**CEGMA (248 proteins)**

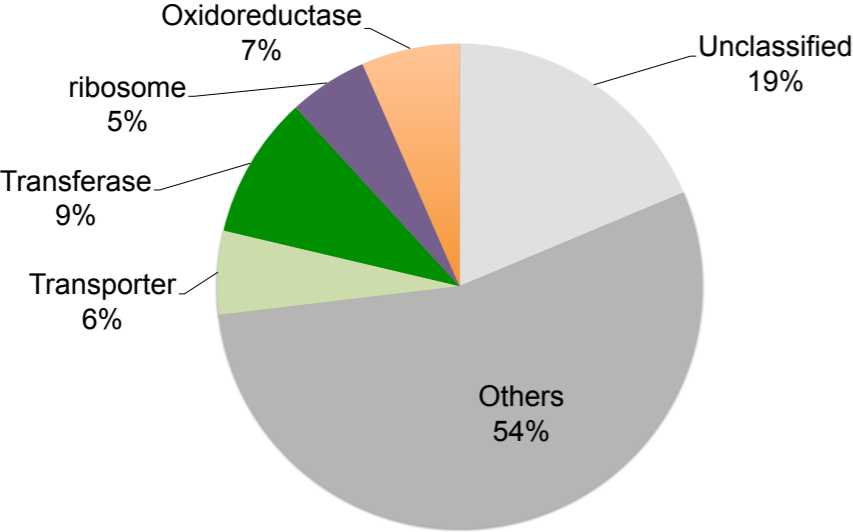

**FGMP (593 proteins)**

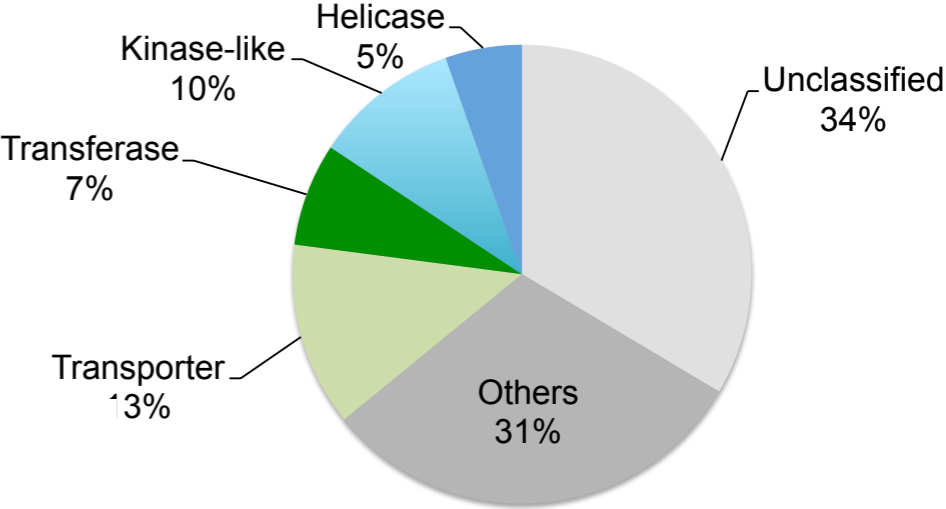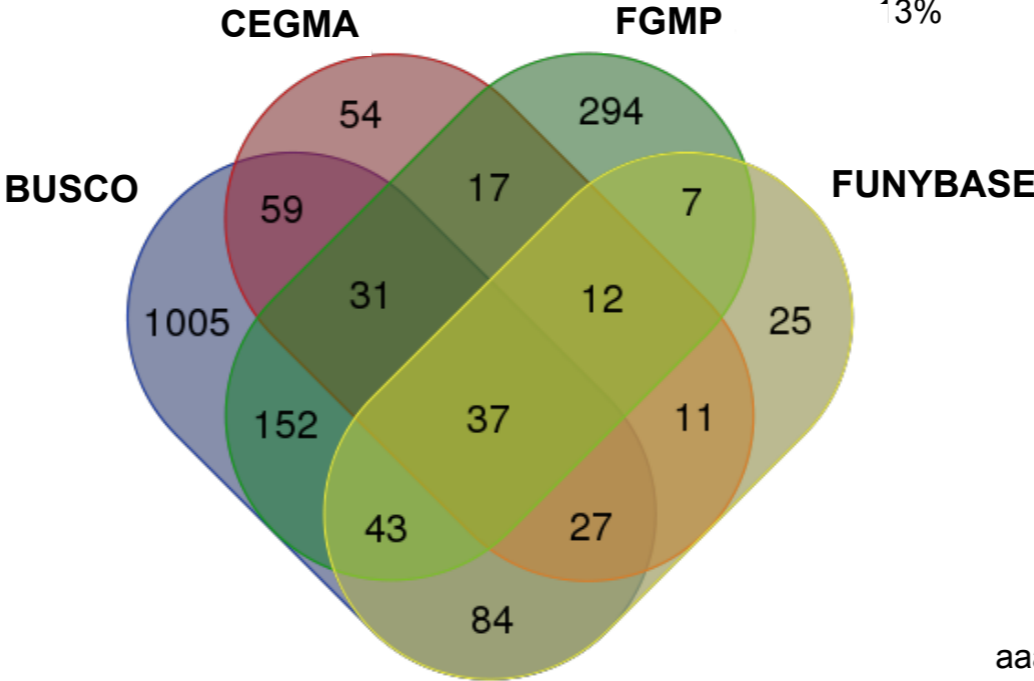

**BUSCO fungi (1,439 proteins)**

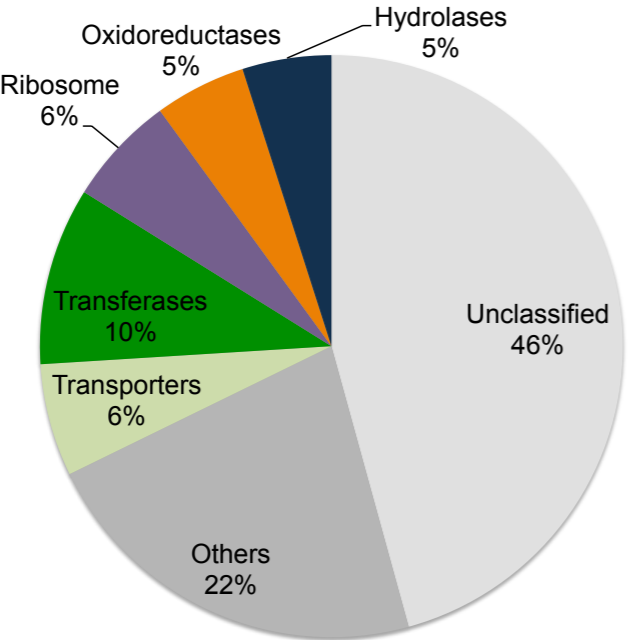

**FUNYBASE (246 proteins)**

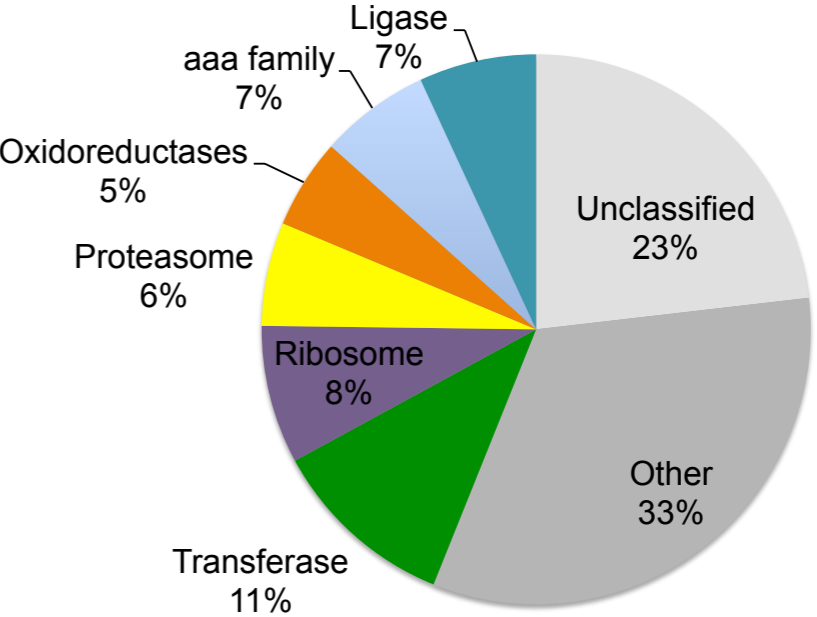

Supplement: Supplementary file 3 — Comparison of protein markers used for genome completeness estimation. (PDF 231 kb) [file 12859_2019_2782_MOESM3_ESM.pdf]

**FGMP\_HCE**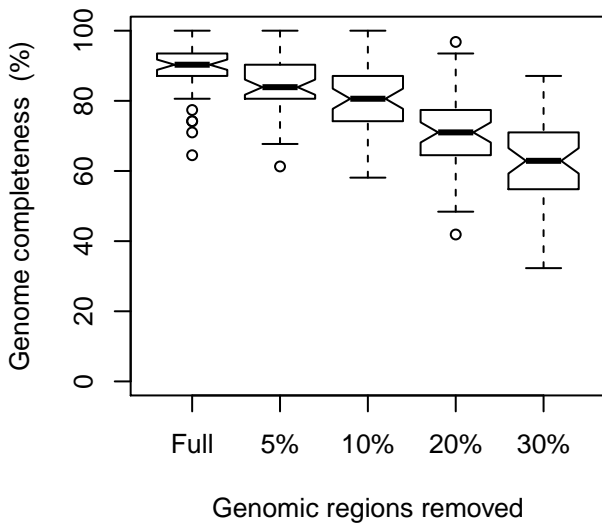**FGMP\_PROT**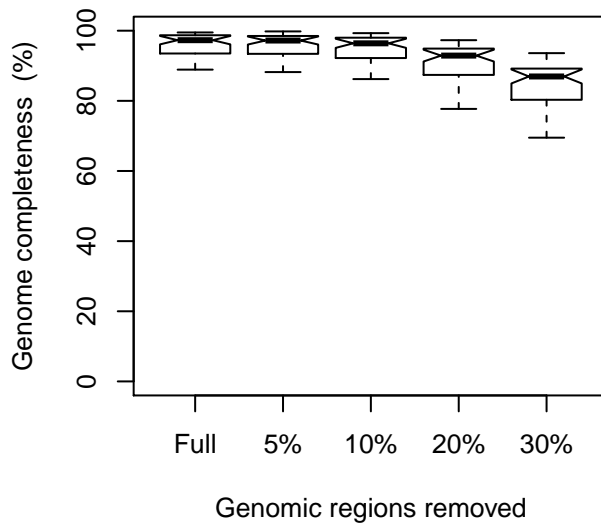**BUSCO (fungi)**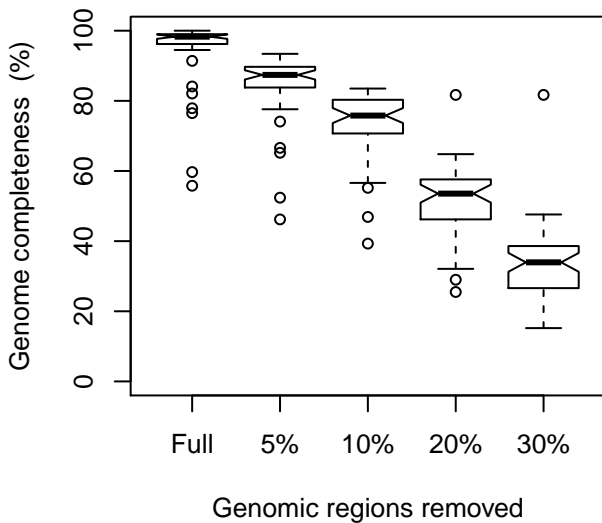**CEGMA**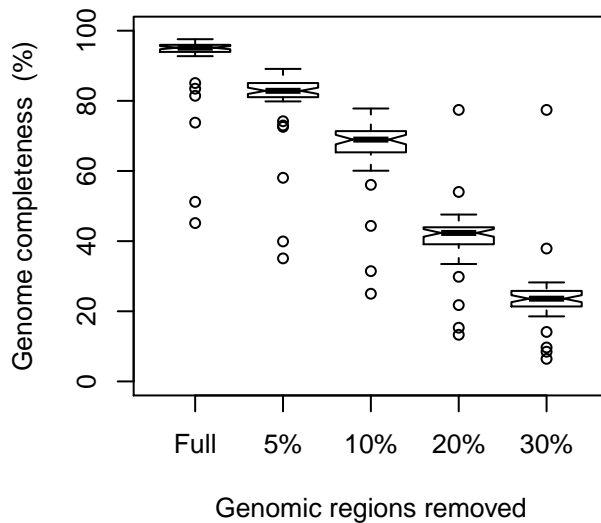

Supplement: Supplementary file 4 — Assessment of genome completeness in 57 fungal genome assemblies. b Plots of genome completeness in 57 fungal genome assemblies. (ZIP 61 kb) [file 12859_2019_2782_MOESM4_ESM.zip › Additional_file_4b.pdf]

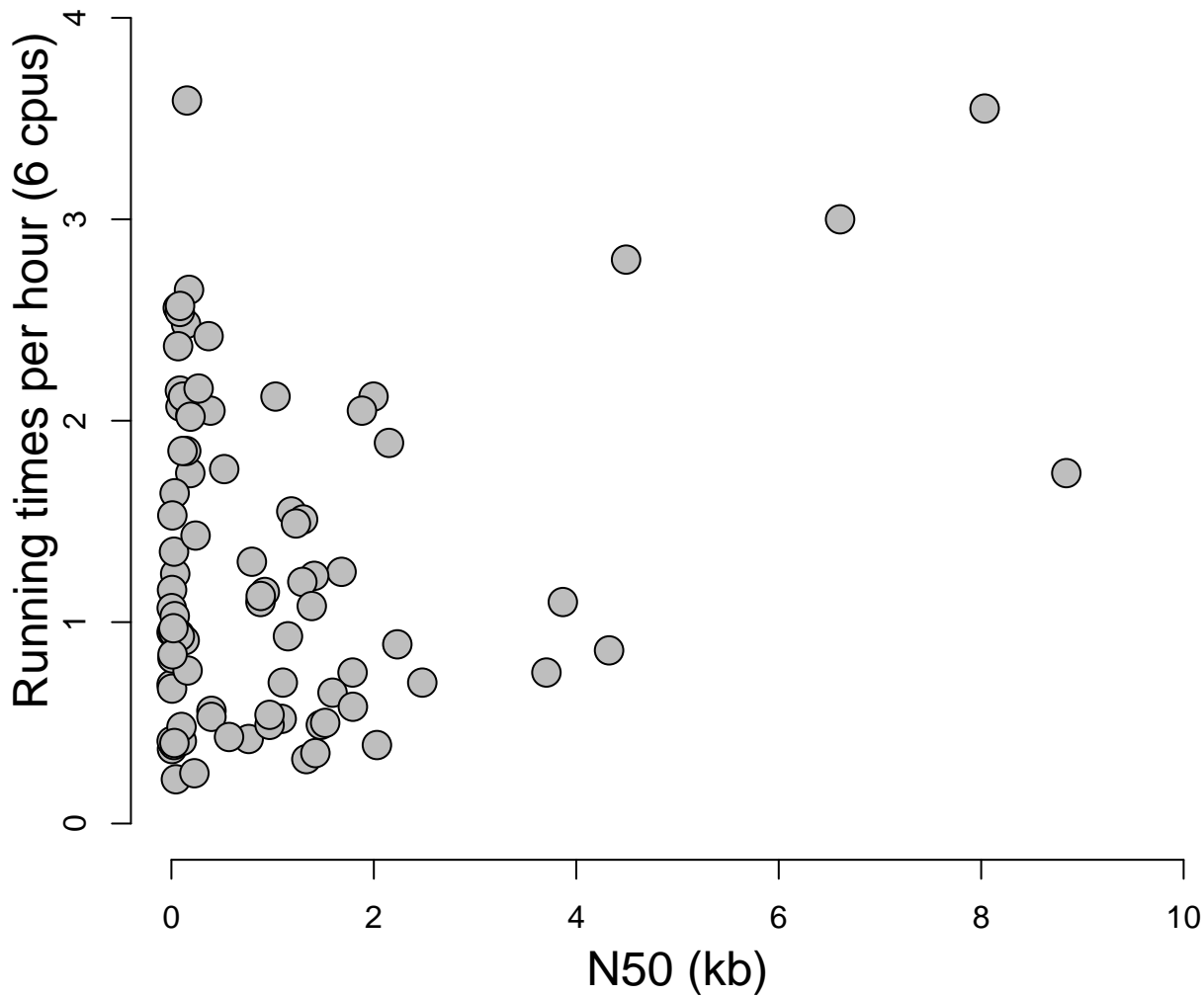

Supplement: Supplementary file 7 — Scatterplot showing the relationship between FGMP running times and the level of fragmentation for different genome assemblies expressed as N50. (PDF 5 kb) [file 12859_2019_2782_MOESM7_ESM.pdf]
